# Urinary Marinobufagenin in Patients with Non-Advanced Chronic Kidney Disease: A Cross-Sectional Study

**DOI:** 10.3390/medicina59081392

**Published:** 2023-07-29

**Authors:** Davide Bolignano, Marta Greco, Mario D’Agostino, Paola Cianfrone, Loredana Tripodi, Roberta Misiti, Mariateresa Zicarelli, Ludovica Ganino, Daniela Patrizia Foti, Michele Andreucci, Giuseppe Coppolino

**Affiliations:** 1 Nephrology and Dialysis Unit, Magna-Graecia University Hospital, 88100 Catanzaro, Italyloredana.tripodi@live.it (L.T.);; 2 Department of Medical and Surgical Sciences, Magna-Graecia University, 88100 Catanzaro, Italy; 3 Clinical Pathology Lab., Magna-Graecia University Hospital, 88100 Catanzaro, Italy; 4 Department of Health Sciences, Magna-Graecia University, 88100 Catanzaro, Italy; 5 Department of Experimental and Clinical Medicine, Magna-Graecia University, 88100 Catanzaro, Italy

**Keywords:** marinobufagenin, chronic kidney disease, biomarker

## Abstract

*Background and Objectives*: The global prevalence of chronic kidney disease (CKD) is on the rise, posing important challenges for healthcare systems. Thus, the search for new factors potentially involved in the pathogenesis, progression and complications of early CKD remains urgent. Marinobufagenin (MBG) is a natriuretic endogenous cardiotonic steroid, and increased circulating levels of it may accelerate kidney damage. In this study, we explored the possible clinical significance of measuring urinary marinobufagenin (uMBG) in patients with non-advanced CKD. *Materials and Methods*: One hundred and eight adult CKD patients (mean age 71.6 ± 10 years, 70.4% male; mean eGFR 40.54 ± 17 mL/min/1.73 m^2^) were enrolled in this cross-sectional study. uMBG was measured together with a series of clinical, anthropometric, laboratory and instrumental analyses. Twenty-five healthy matched subjects served as controls for the uMBG measurement. *Results*: The uMBG values were lower in the patients with CKD as compared to those of the controls (0.37 [IQR: 0.25–0.45] vs. 0.64 [0.46–0.78] nmol/L. *p* = 0.004), and a significant trend in eGFR levels was noticed across the decreasing uMBG tertiles (*p* = 0.03). Regarding the correlation analyses, the uMBG values remained robustly associated with the eGFR in multivariate models employing either uMBG or eGFR as the dependent variable (β = 0.248; *p* = 0.01 and β = 0.139; *p* = 0.04, respectively). Besides the eGFR, the independent predictors of uMBG values in this population were the use of statins (β = −0.326; *p* = 0.001), the presence of diabetes (β = 0.243; *p* = 0.009) and urine sodium (β = 0.204; *p* = 0.01). *Conclusions*: Reduced uMBG excretion may reflect impaired renal clearance, which may contribute to the detrimental effects attributed to this hormone due to systemic accumulation. Future studies are needed to clarify the biological mechanisms placing uMBG at the crossroad of sodium intake and the presence of diabetes in CKD-suffering individuals and to verify whether a statin treatment may somewhat limit the detrimental effects of MBG in the presence of impaired renal function.

## 1. Introduction

Nowadays, the prevalence of chronic kidney disease (CKD) has reached pandemic proportions, posing a significant burden across health care systems at the global level [1]. CKD portends an increased cardiovascular risk, which parallels the severity of renal function impairment, with it being the highest among individuals with end-stage kidney disease (ESKD) undergoing chronic dialysis [2]. Greater research efforts are thus needed to improve the understanding of CKD pathophysiology, to discover alternative therapeutic targets, as well as to identify novel biomarkers to improve cardio-renal risk prediction, particularly in the earlier stages of disease.

Marinobufagenin (MBG) is an endogenous cardiotonic steroid (CTS) which acts by inhibiting the ubiquitous Na^+^/K^+^-ATPase membrane pump, triggering the activation of specific ionic intracellular pathways [3]. The acute effects of MBG include the regulation of the sodium and volume balance, peripheral vasoconstriction, and the enhancement of cardiac inotropism [4]. Accordingly, the blood levels and urine excretion of MBG are increased in experimental models of high salt intake and volume expansion; by the same token, elevated circulating MBG levels has been found among individuals with essential hypertension, congestive heart failure and pregnancy disorders [5].

The kidney plays a key role in maintaining systemic MBG balance by regulating urinary excretion [3]. Hence, not surprisingly, MBG accumulates in the blood of anuric individuals necessitating chronic dialysis [6,7,8], while in kidney transplant recipients, higher circulating MBG levels be associated with the severity of the graft function impairment [9].

Of note, chronic experimental MBG administration promotes fibrosis in the kidney, an effect that could be attenuated by selective anti-MBG antagonists [10], while, in humans, the reversal of renal ischemia due to renal artery angioplasty leads to a parallel decrease in MBG levels in the blood [11]. Hence, more than being regarded as a simple epiphenomenon, higher circulating MBG levels have been looked upon as accelerators of renal damage, and blood MBG measurements in CKD might impart additional information for risk stratification [12].

Changes in urinary MBG excretion (uMBG) are strongly influenced by dietary sodium intake and may reflect pathological cardiac and vascular remodeling in hypertensive individuals [13,14,15]. Yet, to date, evidence is lacking regarding the impact of reduced renal function on uMBG levels, particularly in non-advanced CKD; this hampers a definite interpretation of the role of this hormone in the setting of renal damage and its validity as a biomarker of disease severity.

Therefore, with this information in mind, we have designed a pilot, cross-sectional, proof-of-concept study for the first time to evaluate the possible clinical significance of uMBG measurement in a small cohort of individuals with non-advanced CKD in relation to the severity of renal impairment, the presence of co-morbidities and other clinical factors that could impact the MBG balance.

## 2. Materials and Methods

### 2.1. Patients’ Selection

Three-hundred and six consecutive adult individuals with CKD attending an outpatient clinic in the University Hospital of Catanzaro, Italy, were screened to enter this pilot, observational, cross-sectional study. The main inclusion criteria were having an age >18 years, the presence of mild-to-moderate CKD (NFK stages 2–4; CKD-Epi eGFR <90 and >15 mL/min/1.73 m^2^) and stable renal function with no documented transitory or permanent decrease in the eGFR (≥25% from values recorded at the previous visit) over the 6 months preceding the study. Infections, cancer, recent cardiovascular events requiring hospitalization, active inflammatory states, peripheral oedema, uncontrolled hypertension, severe proteinuria (>3 g/24 h) or having previously undergone kidney transplantation represented the main exclusion criteria. The study was approved by the Local Institutional Review Board, and all participating subjects provided written informed consent.

### 2.2. Clinical Assessment

A complete baseline assessment was performed on every participant before starting the planned outpatients visits. Clinical, demographic and anthropometric parameters were recorded on a standardized, electronic case report form. The patients’ history and medical therapy information was carefully collected via interview and confirmed by the checking patients’ records. Their blood pressure was measured three times at rest, and the average value was recorded for analysis. Laboratory parameters were measured for all the patients according to the standard methods used in the clinical routine.

### 2.3. Urine MBG (uMBG) Measurement

Urine samples were collected on the second morning and were centrifuged at 1227 g for 15 min at 4 °C. Aliquots were immediately stored at −80 °C until they were thawed for batch analysis. MBG was measured in the urine specimens (uMBG) using an ELISA commercially available kit (BlueGene Biotech, Shanghai) following the manufacturer’s instructions. All specimens were often diluted to obtain a concentration for the optimal density according to the ELISA kit instruction. Enzymatic reactions were quantified in an automatic microplate photometer. The measurements were taken blind and in duplicate, and the levels are expressed as nmol/L. To minimize the potential influence of urine dilution, all data analyses were repeated after the normalization of uMBG for urinary creatinine. Twenty-five healthy subjects with conserved renal function, matched for age and sex with the CKD patients, served as the controls for uMBG measurement. The control subjects underwent a low-sodium-level diet (<2300 mg/24 h) during the day before the study to minimize the potential bias on the MBG balance.

### 2.4. Statistical Analysis

Statistical analysis was performed using the SPSS package (version 24.0; IBM corporation), MedCalc Statistical Software (version 14.8.1; MedCalc Software bvba) and the GraphPad prism package (version 8.4.2, GraphPad Software, San Diego, CA, USA). Data are shown as mean ± SD, median [IQ range] or frequency percentage, as appropriate. Differences between groups were assessed using the unpaired t-test for normally distributed values, the Mann–Whitney U test for non-parametric values and the chi-square test, followed by a Fisher’s exact test for frequency distributions.

Differences in clinical parameters across the tertiles of uMBG were assessed using ANOVA for continuous covariates and chi-square tests for categorical data (p for trend). Pearson (R) and the Spearman (Rho) correlation coefficients were employed to test the correlations between variables, as appropriate. Before testing the correlations, all the values showing a skewed distribution were log transformed to better approximate normal distributions. Multiple regression analyses were performed to assess the independent relationships first by employing fully adjusted models including all univariate correlates of uMBG values, respectively. The variables included in the equations (e.g., serum creatinine/eGFR) were not tested in the models, including the corresponding formulas to avoid co-linearity. Stratified models were also built by removing single covariates to explore possible confounding. Data are expressed as partial correlation coefficients (β) and *p* value. All results are considered to be significant for *p* values ≤ 0.05.

## 3. Results

### 3.1. Clinical Characteristics and uMBG Measurement in the Study Cohort

The final study population consisted of 108 adult patients (mean age 71.6 ± 10 years, 70.4% male) who met the inclusion/exclusion criteria for being eligible to participate. Mean eGFR (CKD-Epi) was 40.54 ± 17 mL/min/1.73 m^2^. The CKD etiology was diabetic kidney disease in half of the population, nephroangiosclerosis in 16.7%, glomerulonephritides in 12.9%, interstitial diseases in 7.4% and rare diseases, including ADPKD, in the remaining 3.7%. The prevalence of cardiovascular comorbidities spanned from 91.6% (hypertension) to 5.6% (history of stroke). Diabetes was present in 54.6% of the individuals. Almost all the patients were undergoing combined anti-hypertensive therapy, which frequently included an RAS blocker (82.4%). Roughly one half of the patients underwent diuretic therapy, while the majority were on statins (64.8%) and hypouricemic agents (74.1%).

**Figure 1:**
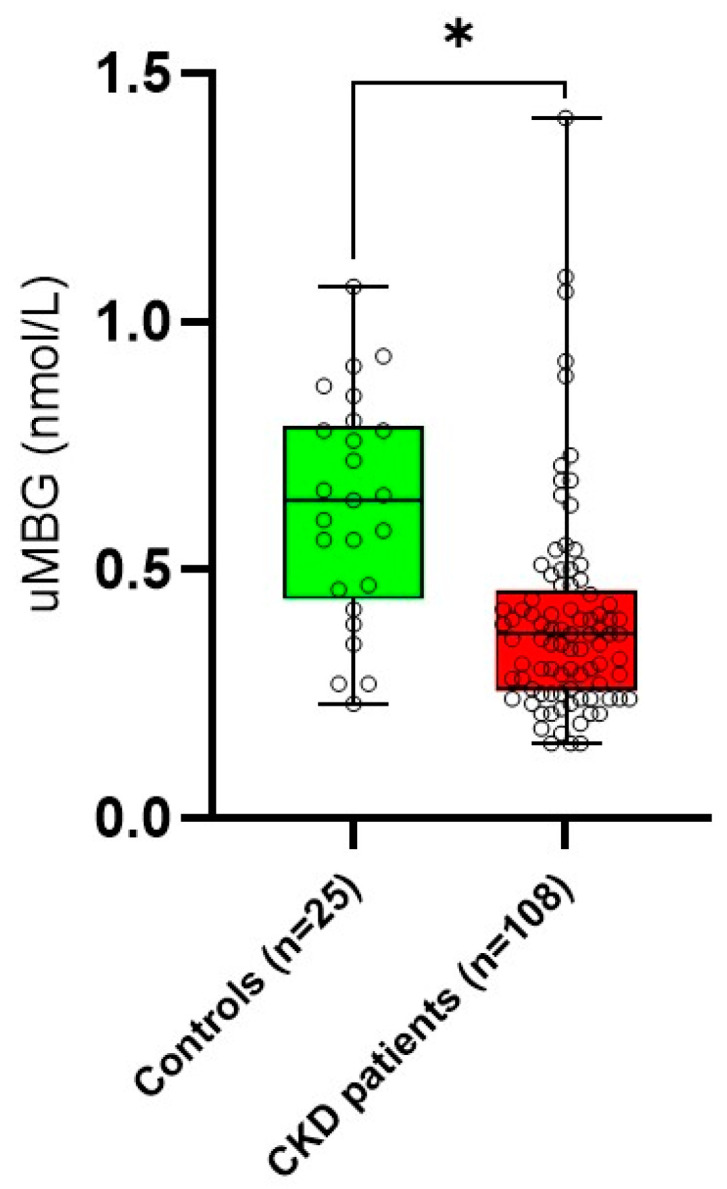
Difference in median urine marinobufagenin (uMBG; nmol/L) between healthy controls and CKD patients. * *p* = 0.004.

The uMBG values in the CKD population were significantly lower compared with those measured in the healthy controls (0.37 [IQR: 0.25–0.45] vs. 0.64 [0.46–0.78] nmol/L. *p* = 0.004 (Figure 1)). In the patients categorized for tertiles of uMBG excretion, growing trends across increasing uMBG values were described for BMI (*p* = 0.01), eGFR (*p* = 0.03), hemoglobin (*p* = 0.03), proteinuria (*p* = 0.04), urine sodium (*p* = 0.03) and urine potassium (*p* = 0.04). The patients with higher uMBG excretion were also younger (*p* = 0.01), more frequently diabetics (*p* = 0.004), less likely to be on statin therapy (*p* = 0.001) and displayed lower levels of urea (*p* = 0.05) and iPTH (*p* = 0.04).

The complete information regarding the characteristics, co-morbidities and active therapy for all the CKD patients and individuals stratified for uMBG tertiles are displayed in Table 1.

### 3.2. Clinical Correlates of uMBG in CKD Patients

**Figure 2:**
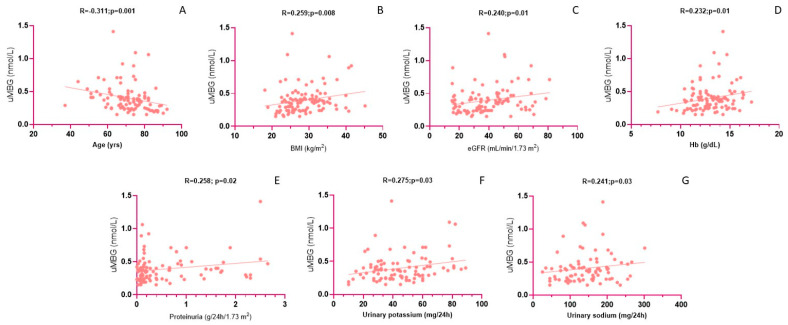
Univariate correlations between uMBG (nmol/L) and age (**A**), BMI (**B**), CKD-Epi eGFR (**C**), hemoglobin (Hb) (**D**), proteinuria (**E**), urinary potassium (**F**) and urinary sodium (**G**). All Pearson correlation coefficients and *p* values are referred to analyses employing log-transformed uMBG values.

In univariate analyses, uMBG was directly associated with BMI (R = 0.259; *p* = 0.008), eGFR (R = 0.240; *p* = 0.01), hemoglobin (R = 0.232; *p* = 0.01), proteinuria (R = 0.258; *p* = 0.02), urine potassium (R = 0.275; *p* = 0.03), urine sodium (R = 0.241; *p* = 0.03) and the presence of diabetes (Rho = 0.211; *p* = 0.03), while the inverse predictors were age (R = −0.311; *p* = 0.001) and the use of statins (Rho = −0.256; *p* = 0.009). In a fully adjusted multivariate model including all the univariate correlates, only the use of statins (β = −0.326; *p* = 0.001), the presence of diabetes (β = 0.243; *p* = 0.009), eGFR (β = 0.248; *p* = 0.01) and urine sodium (β = 0.204; *p* = 0.01) remained significantly associated with uMBG excretion, while the correlations with BMI, urine potassium, age, hemoglobin and proteinuria were lost. Of note, forcing diuretic use (any type) in the model did not affect the relationship between uMBG and urine sodium. The fully adjusted model held considerable power, explaining the 33% of the total uMBG variability in this cohort. Interestingly, the correlations with urine potassium (β = 0.118; *p* = 0.04) and hemoglobin (β = 0.109; *p* = 0.04) reattained statistical significance in an exploratory model excluding eGFR (R^2^ = 28%; *p* < 0.001), suggesting a likely confounding effect of residual renal function on such univariate relationships. By the same token, significant associations between uMBG and, respectively, BMI (β = 0.289; *p* = 0.03) and proteinuria (β = 0.302; *p* = 0.04) re-emerged only in a separate model excluding diabetes (R^2^ = 34%; *p* = 0.008), thereby indicating a significant modulating effect of this disorder. Table 2 and Figure 2 provide detailed results from the correlation analyses of uMBG as a reference variable.

## 4. Discussion

In this cross-sectional study, we explored the clinical significance of urinary marinobufagenin measurements in patients with non-advanced chronic kidney disease. Although they are preliminary, various findings from our study deserve, in our opinion, a focused discussion.

First of all, the uMBG levels found in the CKD patients were, on average, significantly lower compared with those measured in the healthy controls. Hence, renal function impairment might somewhat influence uMBG excretion; in line with this view, the correlation analyses demonstrated a close association between uMBG and eGFR, and such a relationship remained independent from potential confounders in a robust multivariate regression model employing uMBG as the dependent variable.

The observational nature of our study prevents us from clarifying the biological mechanisms underlying the association found between uMBG excretion and the severity of CKD. Under physiological conditions, MBG is released from the adrenocortical glands in response to various stimuli, including volume expansion, sodium overload and RAS activation [3]. MBG is freely filtered into urine, but the excretion mechanism, overall clearance and peripheral metabolism could be largely affected by the presence of a kidney impairment. Increased blood MBG levels have already been reported in anuric individuals undergoing chronic dialysis treatment [6,7,16], while in kidney transplant recipients with partially impaired renal function, higher blood MBG levels were inversely related to residual eGFR and predicted adverse renal outcomes [9]. By the same token, the blood MBG levels were increased in another small CKD cohort [17], while in hypertensive individuals with conserved renal function, higher MBG levels were associated with a more rapid eGFR decline over time [18]. All these observations may suggest a systemic accumulation of MBG following reduced renal excretion; yet, unfortunately, definite conclusions on such a topic cannot be drawn due to the lack of simultaneous measurements of MBG in either blood or urine.

In individuals with arterial hypertension [18], the plasma MBG levels strongly correlated with the severity of albuminuria and proteinuria. In experimental models of human pre-eclampsia [19], this association may reflect a pivotal contribution of MBG to renovascular damage, which may pave the way for using MBG measurements as a surrogate indicator of kidney injuries. Partially in line with these findings, we found a direct correlation between uMBG and proteinuria in the univariate analyses. However, in this cohort, such a relationship was largely confounded by the presence of diabetes, as it remained independent only in the exploratory models that were not adjusted for this co-morbidity. By the same token, we also observed a direct association between uMBG and BMI, which, again, was nullified in the multivariate regression models including diabetes.

Taken all together, these observations may suggest that diabetes can somewhat impact upon uMBG excretion. This hypothesis is further supported by the increasing trend in diabetes prevalence found across growing tertiles of uMBG, and previous evidence indicates that endogenous cardiotonic steroids might be causally involved in the pathogenesis of this disorder [20,21].

Importantly, diabetic kidney disease (DKD) accounts for more than one half of CKD cases in the present cohort, an observation which reflects epidemiological data that rank diabetes as the leading cause of CKD in the developed world [22].Yet, despite such a high prevalence, diabetes remained a robust predictor of uMBG values even in fully adjusted multivariate models, and the strength of the association between uMBG and diabetes is only marginally influenced by residual renal function (β = 0.243 vs. 0.254 in multivariate models with or without eGFR adjustment, respectively).

Two more findings from our study are worth mentioning.

First, the uMBG values in the CKD individuals paralleled the urinary sodium excretion findings, and such a close relationship remained apparently unaffected by possible confounders, including the use of diuretics. This observation pairs well with previous experimental [23] and clinical evidence [15,16] and presumably relies on the natriuretic action of MBG, whose systemic release is mostly caused by a high-level sodium diet [24]. As it is well acknowledged, urinary sodium excretion largely reflects the daily sodium intake. In this view, short-term changes in uMBG could serve as an additional tool for the patient’s monitoring compliance to dietary sodium prescriptions in this high-risk setting. On the other hand, we found a similar, although weaker, relationship with urine potassium excretion, which, however, appeared to be largely confounded by the severity of renal function impairment.

Second, and no less important, the clear influence of statin treatment on uMBG excretion has emerged, as revealed by the significant, decreasing trend in the percentage of statin users across the growing categories of uMBG excretion. In the fully adjusted multivariate regression model, such an inverse association was apparently not even affected by diabetes, ranking as the strongest correlation among all those reported (β = −0.326; *p* = 0.001). Despite the fact that the impact of possible unknown confounders on this relationship cannot be excluded, taken together, these observations may indicate that a statin treatment, by negatively modulating cholesterol metabolism, could also impact upon the biosynthesis or peripheral metabolism of MBG, eventually leading to reduced urinary elimination. In line with this hypothesis, in previous experimental evidence, HMG CoA reductase inhibitors were found to interfere with the sarcolemmal Na+/K+ pump function and to exert an anti-arrhythmogenic effect by modulating the expression and activity of endogenous cardiotonic steroids [25,26].

We acknowledge some key limitations of our study. First, despite the study being powered enough to perform large multivariate adjustments without model overfitting occurring, the observational design cannot rule out the presence of a selection bias or residual confounding, and this prevents us from raising definite assumptions about the biological mechanisms underlying the relationships found between uMBG and the different clinical variables. Last, given the lack of a longitudinal phase, we cannot evaluate whether fluctuations in uMBG over time pair with the changes in renal function, thereby limiting the potential applicability of this substance as a biomarker of kidney damage.

## 5. Conclusions

Individuals with mild-to-moderate CKD display reduced urinary marinobufagenin levels, which parallel the severity of renal impairment. Future studies on larger, heterogeneous populations are advocated to generalize our findings and to shed light on the biological mechanisms underlying the complex interplay between uMBG excretion, diabetes, sodium intake and the use of statins in the presence of impaired renal function.

## Figures and Tables

**Table 1:** Main characteristics of the whole cohort and in patients stratified for tertiles of uMBG (nmol/L). Statistical differences across uMBG strata (*p* for trend) are highlighted in bold.

	All CKD n:108	uMBG <0.29 n:36	uMBG 0.29–0.41 n:36	uMBG >0.41 n:36	p for Trend
**Age (yrs)**	**71.6 ± 10**	**76 ± 8.8**	**71.6 ± 9.5**	**67 ± 10**	**0.01**
Male sex (%)	70.4	69.4	72.2	69.4	0.92
Current smoking (%)	4.2	0	2.8	8.3	0.16
**Diabetes (%)**	**54.6**	**27.7**	**61.1**	**75**	**0.004**
Heart failure (%)	13.9	13.8	19.4	8.3	0.78
Ischemic heart disease (%)	30.6	22.2	36.1	33.3	0.66
Stroke (%)	5.6	11.1	2.8	2.8	0.85
Peripheral Vasculopathy (%)	21.3	27.8	13.9	22.2	0.88
Hypertension (%)	91.6	86.1	97.2	91.7	0.36
CKD etiology					
**-DKD**	**50**	**27.8**	**50**	**72.2**	**0.01**
-Nephroangiosclerosis	16.7	22.2	11.1	16.7	0.70
-GNs	12.9	13.9	13.9	11.1	0.86
-Interstitial	7.4	5.5	8.3	8.3	0.72
-Rare/ADPKD	3.7	2.8	2.8	5.5	0.66
Anti-hypertensive therapy					
-ACEi/ARBs (%)	82.4	80.5	80.5	86.1	0.82
-Beta-blockers (%)	56.5	41.7	44.4	83.3	0.08
-CCBs (%)	44.4	33.3	66.7	33.3	0.22
-Aldosterone antagonists (%)	5.6	11.1	2.8	2.8	0.68
-Frusemide (%)	46.3	50	33.3	55.5	0.47
-Other diuretics (%)	9.3	5.5	2.8	19.4	0.21
ESAs (%)	13.9	19.4	11.1	11.1	0.79
**Statins (%)**	**64.8**	**86.1**	**69.4**	**38.9**	**0.001**
Hypouricemic agents (%)	74.1	55.5	83.3	55.5	0.22
**BMI (Kg/m^2^)**	**28.7 ± 5.3**	**25.6 ± 5.1**	**28.5 ± 5.1**	**30.1 ± 5.7**	**0.01**
Systolic BP (mmHg)	131.2 ± 19.2	134.6 ± 20.8	132.1 ± 20.2	126.4 ± 15.2	0.12
Diastolic BP (mmHg)	72.7 ± 10.3	71.1 ± 10.2	74.2 ± 10.8	72.1 ± 9.8	0.44
**CKD-Epi eGFR (mL/min/1.73 m^2^)**	**40.54 ± 17**	**34.5 ± 14.6**	**41.4 ± 15.4**	**44.6 ± 17.9**	**0.03**
Serum Creatinine (mg/dL)	1.8 ± 0.71	1.90 ± 0.64	1.78 ± 0.69	1.73 ± 0.78	0.18
Urea (mg/dL)	76.9 ± 37.1	85.3 ± 37.5	74.9 ± 28.7	74.3 ± 45	0.05
Glycemia (mg/dL)	111.2 ± 23	109 ± 17	114.8 ± 28.1	108.6 ± 20.6	0.77
Albumin (g/dL)	4.35 ± 0.45	4.34 ± 0.55	4.35 ± 0.38	4.36 ± 0.42	0.82
Serum Sodium (mmol/L)	140.4 ± 3.4	139.5 ± 3.9	140.2 ± 3.3	141.3 ± 2.9	0.18
Serum Potassium (mmol/L)	4.75 ± 0.61	4.64 ± 0.64	4.83 ± 0.56	4.68 ± 0.32	0.65
Serum Calcium (mg/dL)	9.52 ± 0.53	9.40 ± 0.51	9.69 ± 0.45	9.46 ± 0.55	0.58
Serum Phosphate (mg/dL)	3.5 ± 0.73	3.52 ± 0.52	3.56 ± 0.56	3.60 ± 0.87	0.23
Serum Magnesium (mg/dL)	2.09 ± 0.38	1.93 ± 0.38	2.23 ± 0.31	1.83 ± 0.35	0.54
Red blood cells (n×10^6^)	4.5 ± 0.9	4.46 ± 1.2	4.69 ± 0.7	4.50 ± 1.02	0.68
**Hemoglobin (g/dL)**	**12.9 ± 1.7**	**12.4 ± 1.9**	**13.1 ± 1.6**	**13.3 ± 1.7**	**0.03**
Platelets (n×10^3^)	223.8 ± 69	229.4 ± 69.9	214 ± 70.3	231 ± 67.3	0.52
Total Cholesterol (mg/dL)	141 ± 32.6	138.7 ± 16.7	142.6 ± 30.2	140.3 ± 38.2	0.48
LDL Cholesterol (mg/dL)	73 ± 30.6	67 ± 21.7	74.5 ± 28.7	73.4 ± 38.6	0.73
Triglycerides (mg/dL)	103.5 [71.5–176]	145.5 [86.2–243]	94.5 [63.2–102.7]	111.5 [74.7–146]	0.59
**iPTH (pg/mL)**	**89.2 [52.7–159.5]**	**135.4 [55.9–191.4]**	**91.8 [51.7–126.8]**	**75.2 [50.7–158]**	**0.04**
Uric Acid (mg/dL)	5.4 ± 1.6	5.31 ± 1.46	5.48 ± 1.74	5.35 ± 1.76	0.86
Creatinuria (mg/dL)	11.5 [9.4–16]	11.1 [3.1–13.2]	11.4 [9.4–17.2]	12.7 [8.9–16.8]	0.37
**Proteinuria (g/24 h/1.73 m^2^)**	**0.287 [0.124–1.035]**	**0.14 [0.07–0.45]**	**0.19 [0.10–0.65]**	**0.25 [0.13–1]**	**0.04**
**Urine sodium (mg/24 h)**	**150.9 ± 58.9**	**133 ± 62.7**	**139.9 ± 50.9**	**170.3 ± 73**	**0.03**
**Urine potassium (mg/24 h)**	**49.3 ± 16.8**	**43.4 ± 9.1**	**49.5 ± 18.8**	**54.7 ± 18.3**	**0.04**
**uMBG (nmoL/L)**	**0.37 [0.25–0.45]**	**0.23 [0.21–0.24]**	**0.37 [0.31–0.39]**	**0.52 [0.47–0.71]**	**<0.001**

Legend: CKD: chronic kidney disease; eGFR: estimated glomerular filtration rate; GNs: glomerulonephritis; ADPKD: autosomal polycystic kidney disease; ACEi: ACE inhibitors; ARBs: angiotensin receptor blockers; CCBs: calcium channel blockers; ESAs: erythropoiesis stimulating agents; BMI: body mass index; BP: blood pressure; LDL: low-density lipoprotein; IPTH: intact parathyroid hormone; uMBG: urine marinobufagenin.

**Table 2:** Univariate and multiple correlation analysis of (log) uMBG. Statistically significant associations at multivariate analyses are highlighted in bold.

	Univariate Correlation Coefficient	*p*
Age	−0.311	0.001
Statins	−0.256	0.009
BMI	0.259	0.008
eGFR	0.240	0.01
Hemoglobin	0.232	0.01
(log)Proteinuria	0.258	0.02
Urine potassium	0.275	0.03
Urine sodium	0.241	0.03
Diabetes	0.211	0.03
	**Multivariate standardized correlation coefficient (β)**	***p***
Model 1: Fully adjusted		
**Statins**	**−0.326**	**0.001**
**Diabetes**	**0.243**	**0.009**
**eGFR**	**0.248**	**0.01**
**Urine sodium**	**0.204**	**0.01**
BMI	0.151	0.09
Urine potassium	0.093	0.28
Age	−0.059	0.54
Hemoglobin	0.058	0.54
(log)Proteinuria	0.049	0.59
Model 2: Excluding eGFR		
**Diabetes**	**0.254**	**0.006**
**Statins**	**−0.380**	**0.01**
**Urine sodium**	**0.192**	**0.03**
**Urine potassium**	**0.118**	**0.04**
**Hemoglobin**	**0.109**	**0.04**
Age	−0.015	0.66
BMI	0.109	0.54
(log)Proteinuria	−0.012	0.79
Model 3: Excluding Diabetes		
**Statins**	**−0.231**	**0.01**
**BMI**	**0.289**	**0.03**
**(log)Proteinuria**	**0.302**	**0.04**
**eGFR**	**0.274**	**0.05**
**Urine sodium**	**0.196**	**0.05**
Age	0.196	0.18
Urine potassium	0.123	0.34
Hemoglobin	0.034	0.80

Model 1 (fully adjusted): Multiple R = 0.57, R^2^ = 33%; *p* < 0.001; Model 2: Multiple R = 0.53, R^2^ = 28%; *p* < 0.001; Model 3: Multiple R = 0.58, R^2^ = 34%; *p* = 0.008; Legend: BMI: body mass index; eGFR: estimated glomerular filtration rate.

## Data Availability

Raw data from this study can be shared by the corresponding author upon reasonable request.
